# Factors Associated with HIV Related Stigma among College Students in the Midwest

**DOI:** 10.3934/publichealth.2017.4.347

**Published:** 2017-07-05

**Authors:** Caroline Kingori, Mavis Adwoa Nkansah, Zelalem Haile, Kay-Anne Darlington, Tania Basta

**Affiliations:** 1 Department of Social and Public Health, Ohio University, Athens OH 45701; 2 Department of International Development Studies, Ohio University; 3 Department of Social Medicine, Heritage College of Osteopathic Medicine, Ohio University; 4 Department of Communication, University of Rio Grande, Rio Grande OH 45674

**Keywords:** AIDS, college, HIV knowledge, Stigma, sexual health

## Abstract

In general, U.S. college students have low perceived susceptibility of acquiring HIV infection while 15–25 percent of youth have had negative perceptions towards HIV positive individuals. Factors associated with HIV stigma among college students were examined in a convenience sample of 200 students. Descriptive and inferential statistics were utilized to summarize the data. Only four percent of participants responded correctly to HIV transmission knowledge items. HIV transmission knowledge scores were significantly higher for participants who were single with partner and those who resided outside university residential dorms (*p* < 0.05). There was a significant negative correlation between composite HIV knowledge scores and stigma scores r = −0.18 (*p* < 0.05). After adjusting for confounders, a marginal significant negative linear relationship emerged (*β* = −0.09, *p* = 0.06) between HIV knowledge and stigma. HIV prevention education among college students needs to be addressed with nuance to minimize HIV knowledge gaps, stigma and student risk perception that impacts HIV prevention and stigma against those living with HIV.

## 1. Introduction

According to the Joint United Nations Program on HIV/AIDS, approximately 36.9 million people globally were living with HIV while another 1.2 million died from AIDS-related illnesses in 2014 [1]. However, incidence and prevalence rates vary across countries and regions. In the US, HIV incidence rates have remained stable at 50,000 new infections annually. However, there were over 1.2 million people over the age of 13 years old reportedly living with HIV and almost 13 percent of those individuals, were unaware of their HIV status as of 2015 [2]. Additionally, the gaps in HIV knowledge as well as access to information have led to labeling, stereotyping and discrimination of people living with HIV/AIDS (PLWHA) [3].

While there are glaring challenges in the fight against HIV transmission, significant progress in HIV treatment is documented. In 2016, the World Health Organization issued a directive to test and treat all who present with HIV infection regardless of their viral load [4]. However, the cost of HIV treatment has increased over time. As of 2010, the estimated lifetime cost of treating HIV infection was $379,668 [5]. Such information signifies the need to enhance HIV prevention strategies, particularly among those who do not know their HIV status, to minimize spread of infection and bridge the gap between those who have access to HIV knowledge and those who do not.

With regard to awareness of HIV serostatus, reports indicate that about 51 percent of youth (13–24 years old) living with HIV in the US, were not aware of their status in 2012 [6]. Those disproportionately affected include: youth identifying as gay and bisexual (72 percent of incidence rates among youth); youth identifying as African American (57 percent of incidence rates among youth) and; youth identifying as Hispanic/Latino (30 percent of new infections among youth) [7]. Such statistics are a cause for concern and need to be examined with nuance. Targeted contextually relevant interventions are needed among disproportionately affected groups in order to sustain HIV prevention efforts.

US High schools, colleges and universities have designed numerous programs to educate and empower youth to practice safe sex, if not abstinence. Ellis [8] found that 79 percent of college students had attended a prior HIV/AIDS and STD program and 73 percent believed that their knowledge of HIV/AIDS was adequate. Ellis [8] contradicts older findings [9] that indicated that college students scored an average of 78 percent on objective HIV knowledge items, but when asked to subjectively rate their HIV knowledge, 65 percent of participants self-rated their HIV knowledge level (HIV/AIDS in general) as medium, and 50 percent of participants were considered having good knowledge on HIV testing [8]. Gaps in college students' knowledge regarding HIV transmission is also reported in other studies [6],[10],[11].

Such gaps support evidence that shows that U.S. college students may have low perceived susceptibility of acquiring HIV infection while engaging in high-risk sexual behavior [11]–[14]. Brown et al. [12] found that student participants had adequate knowledge of HIV prevention, but only 34 percent consistently practiced safe sex. Furthermore, El Bcheraou et al. [14] found that students who perceived themselves to be of average to high-risk for HIV did not engage in protective intercourse. Similarly, Sutton et al. [11] found that a majority of student participants (64%) had reported inconsistent condom use at their last sexual encounter and had never been tested for HIV. These studies show a troubling trend of low perceived susceptibility to HIV infection among college students and support a nuanced approach to address HIV prevention.

With regard to relationship between HIV knowledge and stigma, Balfour et al [15] found that health science college students and pharmacists in Guyana, South America, had high levels of knowledge, and reported low levels of stigma. Such students had been exposed to HIV knowledge via health science courses, HIV training or were in a health science related major. On the other hand, health science college students as well as pharmacists had some important HIV knowledge gaps on complex treatment issues such as medication adherence and drug resistance [15]. Balfour et al [15] established that although, HIV curriculum has enhanced HIV knowledge among health science college students and pharmacists, gaps in HIV complex issues, such as treatment, remain. Such findings call for a closer look at the role HIV stigma plays in influencing uptake of HIV knowledge and other preventive strategies.

HIV stigma, a socially constructed phenomenon, discredits people who are HIV positive and deems them socially unfit which leads to labeling, stereotyping and discrimination [3],[16],[17]. PLWHA are often stigmatized on multiple levels, particularly if they are from a disenfranchised group such as a sexual (e.g., gay or bisexual men) or gender (e.g., transgender, non-conforming) minority who are known to have increased risk for HIV or injection drug users who have historically been stigmatized and blamed for the spread of the infection [18]. While the stigma against such groups has dwindled, PLWHA still face discrimination. Recent studies acknowledge that participants had negative perceptions towards HIV positive individuals whereby they would not: patronize an HIV positive merchant, attend to a sick relative [10], be comfortable sending a child to school where one of the students was positive, or work in an office with an HIV positive employee [19].

Beyond knowledge on HIV transmission via sexual contact, other studies reported confusion and lack of adequate knowledge on other routes of HIV transmission. For example, some students reported mosquitos could transmit HIV, while others were not aware of medicines that prevent maternal to child transmission of the infection [9],[12]. Such misconceptions of HIV transmission may lead to stigmatizing perceptions and attitudes towards people who are HIV positive.

Even though new HIV infections among youth have stabilized, a large proportion of them are unaware of their HIV status. Therefore, the lack of knowledge about their HIV status, coupled with the misconceptions on HIV transmission among college students, is likely to increase stigma against HIV positive individuals. HIV prevention among college students needs novel strategies. The main purpose of this study was to explore factors associated with HIV related stigma in relation to adequate access to information and HIV knowledge, and to illuminate gaps in HIV prevention strategies.

## 2. Methods

### 2.1. Sample and research design

Participants for this pilot cross-sectional study were recruited from a pool of college students at a university in the Midwest. Approval to carry out this study was obtained from the Institutional Review Board at the institution where data was collected. The convenience sample comprised students recruited from undergraduate and graduate classes by requesting permission from faculty members to administer a pen and paper survey during class sessions. The student population at the time was 40,000. The sample was broadly representative of the racial distribution at the university, which is predominantly non-Hispanic white. Additionally, participants were sought at high-traffic areas of the university campus such as the entrance of the main student center. Students received a $5 gift card for participation in the study. A total of 204 surveys were received. However, because only 4 surveys were received from graduate students, these were excluded from the analyses resulting in a final sample of 200 undergraduate students.

To be eligible to participate in this study, participants had to satisfy the following inclusion criteria: (a) 18 years of age or older; and (b) currently registered at the university. For participants recruited at high-traffic areas, they completed the survey in a private area of the student center after receiving an explanation of the study and signing the consent form. In the classrooms, faculty members allocated the last 15 minutes of class time to collect data. The instructor at the beginning of class made an announcement regarding the study. Research assistants provided a summary of the study to the students and those who wanted to participate were left in the class while those who chose not to participate were allowed to leave the classroom. Each participant read and signed an informed consent form before completing the survey. They were informed of the voluntary nature of the study and their right to discontinue at any point without recourse.

### 2.2. Measures

#### 2.2.1. HIV stigma

The main outcome of interest was HIV stigma. A 14-item HIV stigma instrument [20] was used to measure HIV stigma, via items addressing feelings towards, attitudes and interaction with people living with HIV/AIDS. Previous internal consistency of the ten items from this scale was moderate (Cronbach's *α* = 0.77 to 0.79) [20]. Responses were measured using a four-point scale: *strongly disagree, somewhat disagree, somewhat agree and strongly agree*. This study's questionnaire had a strong internal consistency (Cronbach's *α* = 0.85). Table 1 shows a descriptive summary of the HIV stigma questions. A composite HIV stigma score was created by summing the responses across the different questions and rescaling the result from 0–100 where a higher score indicating higher level of HIV stigma.

**Table 1.** Distribution of HIV/AIDS stigma items (N = 200).

	*“Strongly” or “Somewhat” Agree*		
	*Overall (n = 199)*	*Female (n = 154)*	*Male (n = 45)*
	*%*	*%*	*%*
I feel sympathetic toward individuals living with HIV/AIDS	95.0	96.1	91.1
I feel angry toward individuals living with HIV/AIDS*	4.5	3.3	8.9
I feel afraid of individuals living with HIV/AIDS	32.0	31.8	33.3
I feel disgusted by individuals living with HIV/AIDS	7.5	5.8	13.3
People with HIV/AIDS should be legally separated from others to protect the public health *	5.5	5.8	4.6
The names of people with HIV/AIDS should be made public so that others can avoid them	9.5	7.8	15.6
Women who are pregnant should be required to get tested for HIV in order to protect the health of their unborn baby *	75.0	78.2	65.9
Most people living with HIV/AIDS don't care if they infect others with the virus *	5.6	4.6	8.9
Most people living with HIV/AIDS are responsible for having their disease	30.0	28.6	35.6
People who got HIV/AIDS through sex or drug use have gotten what they deserve	8.5	8.4	8.9
I would be uncomfortable if my child attended school where one of the students was known to be living with HIV/AIDS	26.0	24.7	31.1
I would be uncomfortable working in an office where one of my co-workers was known to be living with HIV/AIDS	14.0	13.0	17.8
I would be uncomfortable shopping at a local grocery store if the owner was known to be living with HIV/AIDS	10.0	10.4	8.9
I would be uncomfortable going to a doctor if he/she was known to be living with HIV/AIDS	27.0	25.9	31.1

* indicates variables with missing values. Missing value: maximum = 4 cases minimum = 1 case.

#### 2.2.2. HIV knowledge

The HIV-KQ 18 knowledge instrument [21] was adapted and used to quantify knowledge of HIV. Research has shown that this instrument's internal consistency is strong and varies across samples (Cronbach's *α* = 0.75 to 0.89) [21]–[24]. In the current study, one item was inadvertently omitted and only 17 items were utilized. Questionnaire items examined knowledge on various HIV transmission routes, myths, treatment and prevention. The responses were: *True, False* and *Don't know*. *Don't know* responses were classified as an incorrect response. The questionnaire in the current study had strong internal consistency (Cronbach's *α* = 0.75). Table 2 presents a descriptive summary of the HIV knowledge questions. A composite HIV knowledge score was created by summing the responses across the different questions and rescaling the result from 0–100 where a higher score indicated higher HIV knowledge.

**Table 2.** Distribution of HIV/AIDS related knowledge (N = 200).

	*Correct Response*		
	*Overall (n = 199)*	*Female (n = 154)*	*Male (n = 45)*
	*%*	*%*	*%*
Coughing and sneezing DO NOT spread HIV	72.0	72.7	68.9
A person can get HIV by sharing a glass of water with someone who has HIV	71.0	72.1	66.7
Pulling out the penis before a man climaxes/cums keeps a woman from getting HIV during sex	90.5	89.5	93.3
A woman can get HIV if she has anal sex with a man *	79.4	77.1	86.7
Showering, or washing one's genitals/private parts, after sex keeps a person from getting HIV	85.5	83.8	91.1
All pregnant women infected with HIV will have babies born with AIDS *	51.5	53.9	44.4
People who have been infected with HIV quickly show serious signs of being infected	83.4	82.4	86.7
There is a vaccine that can stop adults from getting HIV *	63.8	**60.1**	**77.8**^†^
People are likely to get HIV by deep kissing, putting their tongue in their partner's mouth, if their partner has HIV *	65.5	**69.5**	**51.1**^†^
A woman cannot get HIV if she has sex during her period	89.0	**87.0**	**97.8**^†^
There is a female condom that can help decrease a woman's chance of getting HIV *	60.6	59.8	65.9
A natural skin condom works better against HIV than does a latex condom *	39.7	40.26	38.6
A person will NOT get HIV if she or he is taking antibiotic	76.0	72.7	86.8
Having sex with more than one partner can increase a person's chance of being infected with HIV *	91.5	89.5	97.8
Taking a test for HIV one week after having sex will tell a person if she or he has HIV	35.7	38.3	27.3
A person can get HIV from oral sex *	61.4	60.9	62.2
Using Vaseline or baby oil with condoms lowers the chance of getting HIV *	67.8	67.3	71.1

* indicates variables with missing values. Missing value maximum = 3 cases minimum = 1 case; † *p* < 0.05.

### 2.3. Access to HIV information

Access to HIV information was measured based on participants' response to access to HIV related information in the past 30 days from sources that include: (i) poster, signs and billboards; (ii) pamphlets or brochures; (iii) newspaper; (iv) meeting, classes, presentations about HIV; (v) television and/or radio; (vi) healthcare professional; (vii) internet; (viii) social media.

Responses were measured using a 7-point scale: *never, seldom, sometime, and often, somewhat often, frequently* and *very often*. Access to HIV information index was constructed by assigning each of these responses a factor score generated through principal component analysis. These scores were then standardized in relation to a standard normal distribution with a mean of zero and a standard deviation of one. A sum of these scores was created to rank individuals based on their frequency of access to HIV information. Finally, the scores were converted into tertiles from one (low) to three (high) access.

### 2.4. Covariates

The covariates included in the analysis were based on previous literature reporting their influence on HIV stigma. These include age and gender, [3],[25], race/ethnicity [24], relationship status, sexual orientation [3],[26] academic status [25], type of residence and access to HIV information [26].

### 2.5. Statistical analysis

Descriptive and inferential statistics were utilized to summarize the data. The distributions of both composite HIV knowledge and HIV stigma scores were approximately normal. Composite scores of HIV stigma and HIV knowledge were compared between the different age groups using the independent sample-test and analysis of variance (ANOVA) tests. Pearson's correlation coefficient was used to examine the strength of a linear relationship between composite HIV knowledge and HIV stigma scores. Proportions of correct responses to HIV knowledge items and proportions of agreement with HIV stigma items were compared between males and females using the Chi-square (χ^2^) tests.

The crude association between HIV knowledge, access to HIV information, covariates and HIV stigma were obtained using bivariate and multivariable linear regression analysis. Independent variables with *P* values of 0.20 or below in the bivariate analysis were entered simultaneously into a multivariable model. To assess for potential collinearity, regression diagnostics were utilized. The variance inflation factor and tolerance values were all within acceptable limits. Model fit was evaluated through visual inspection of the parameter estimates and standard errors. All statistical analyses were conducted using SAS^®^ 9.4 [27].

## 3. Results

Table 3 presents characteristics of the study sample. Study participants ranged in age from 19–31 years with a mean (standard deviation) of 21.0 (1.68) years. Seventy-seven percent of the study participants were female and 88 percent of the study participants were non-Hispanic White. Twenty percent of the participants were freshmen; 35 percent sophomores; 25 percent juniors and 18 percent seniors.

**Table 3.** Description of sample characteristics by HIV knowledge and stigma scores (N = 200).

		*Overall*		*HIV knowledge composite score*			*HIV stigma composite score*	
		*n (%)*		*Mean (SD)*	*p*		*Mean (SD)*	*p*
*Age*					0.72			0.22
	19–21 years	140 (70.7)		68.52 (19.25)			21.66 (14.91)	
	>21 years	58 (29.3)		71.29 (19.95)			19.12 (12.65)	
*Gender*					0.47			0.03
	Female	154 (77.0)		68.90 (20.79)			19.65 (13.71)	
	Male	45 (22.5)		71.24 (13.63)			25.13 (15.48)	
*Race*					0.47			0.4
	White	176 (88.0)		69.01 (19.41)			20.45 (13.74)	
	Other	24 (12.0)		72.02 (19.17)			23.61 (17.71)	
*Relationship Status*					0.02			0.47
	Single	127 (63.5)		67.25 (20.12)			21.33 (15.28)	
	Single with partner	72 (36.0)		73.28 (17.52)			19.9 (12.42)	
*Type of Residence*					0.03			0.23
	Dorms	113 (56.5)		66.89 (19.95)			21.87 (15.37)	
	Other	83 (41.5)		72.61 (18.18)			19.48 (12.64)	
*Sexual Orientation*					0.19			0.01
	Heterosexual	180 (90.0)		69.15 (19.31)			21.41 (14.20)	
	Other	15 (7.5)		75.68 (11.94)			12.53 (11.85)	
*Academic Status*					0.01			0.14
	Freshman	41 (20.5)		61.40 (21.41)			24.91 (17.20)	
	Sophomore	69 (34.5)		69.39 (18.00)			21.46 (13.82)	
	Junior	50 (25.0)		71.41 (19.23)			19.00 (13.68)	
	Senior	36 (18.0)		74.5 (16.87)			18.32 (11.82)	
*Access to HIV information*					0.29			0.83
	Low	64 (32.0)		66.91 (21.61)			21.42 (16.29)	
	Medium	68 (34.0)		72.22 (18.44)			20.85 (13.12)	
	High	68 (34.0)		68.33 (17.21)			19.95 (13.24)	

An independent-samples t-test indicated that HIV knowledge scores were significantly higher for single with partner than for those identifying as single (*p* < 0.05). Similarly, HIV knowledge scores were significantly higher for students who resided in other type of residence than students who lived in the dorm (*p* < 0.05). There was a main effect of academic status on HIV knowledge (*p* < 0.05). HIV stigma scores were significantly higher for males than for females (*p* < 0.05). Similarly, HIV stigma scores were significantly higher for heterosexual students than for students who reported other sexual orientation (*p* < 0.05) (see Table 3).

Thirty two percent of the participants agreed with the item *“I feel afraid of individuals living with HIV/AIDS”* and 30 percent of the participants agreed with the item *“Most people living with HIV/AIDS are responsible for having their disease”* (Table 1). There were no statistically significant differences in HIV stigma items by gender.

Only four percent of participants responded correctly to all HIV knowledge items. Approximately one-third of the participants correctly responded to the item *“Taking a test for HIV one week after having sex will tell a person if she or he has HIV”* and only 40 percent of the participants correctly responded to the item *“A natural skin condom works better against HIV than does a latex condom.”* Only 50 percent of the participants correctly responded to the item *“All pregnant women infected with HIV will have babies born with AIDS.”* For three HIV knowledge items, the proportion of correct responses differed by gender. Compared to females, greater proportion of males correctly responded to the following items *“There is a vaccine that can stop adults from getting HIV” (p < 0.05) and “A woman cannot get HIV if she has sex during her period”* (*p* < 0.05). On the other hand, compared to males, greater proportion of females correctly responded to the item *“People are likely to get HIV by deep kissing, putting their tongue in their partner's mouth, if their partner has HIV”* (*p* < 0.05) (Table 2).

Examination of the relationship between composite HIV knowledge scores and composite stigma scores revealed that there was a statistically significant negative correlation between composite HIV knowledge scores and stigma scores r = −0.18 (*p* < 0.05). After adjusting for, gender, race/ethnicity, sexual orientation, and academic status, we found a marginal significant negative linear relationship between HIV knowledge and HIV stigma (*β* = −0.09, *p* = 0.06). In the multivariable model other factors that showed a significant linear relationship with HIV stigma include gender and sexual orientation. Compared to males, females had low level of HIV stigma (*β* = −6.31, *p* = 0.01) after adjusting for race/ethnicity, sexual orientation, academic status and composite HIV knowledge score. Similarly, controlling for gender, race/ethnicity, and academic status heterosexual students had higher level of stigma than students with other sexual orientation (*β* = 8.48, *p* < 0.01) (Table 4).

**Table 4.** Association between HIV knowledge and stigma.

		*Unadjusted*				*Multivariable-adjusted*		
		*β*	*SE*	*p*		*β*	*SE*	*p*
*Age*		−1.83	2.35	0.43		NA	-	-
*Gender*								
	Female	−5.96	2.44	0.01		−6.31	2.39	0.01
*Race*								
	White	−4.85	3.30	0.14		−4.56	3.18	0.15
*Relationship status*								
	Single	0.82	2.19	0.70		NA	-	-
*Type of Residence*								
	Dorms	2.10	2.13	0.31		NA	-	-
*Sexual Orientation*								
	Heterosexual	8.37	3.94	0.03		8.48	3.83	0.02
*Academic Status*								
	Freshman	6.33	3.37	0.06		4.73	3.29	0.15
	Sophomore	2.92	3.03	0.33		2.03	2.90	0.48
	Junior	0.67	3.19	0.83		−0.38	3.06	0.89
*Knowledge*		−0.12	0.05	0.02		−0.09	0.05	0.06
*Access to Information*								
	Low	1.39	2.58	0.59		NA	-	-
	Medium	−0.06	2.55	0.97		NA	-	-
*Adjusted R^2^*						0.07		

## 4. Discussion

The current study explored factors associated with HIV related stigma in relation to adequate access to information and HIV knowledge among college students and to illuminate gaps in HIV prevention strategies. University students often engage in high-risk behaviors such as: multiple sexual partners, unprotected vaginal, oral and/or anal sex and alcohol and drug use that put them at risk for HIV infection [10],[11],[28],[29]. It is, therefore, important to assess the level of HIV knowledge and other factors influencing stigma towards PLWHA in order to respond with appropriate educational and skill enhancing interventions. The information gathered in this pilot study can be used to design and tailor HIV outreach and educational activities among college students.

### 4.1. HIV knowledge

Only four percent of the participants correctly answered all 17 items of the HIV knowledge items and the average score for the participants on the HIV knowledge questions was 69.4, which is lower than college students in other studies [11],[30],[31]. To address this challenge, peer educator interventions can help boost the level of HIV knowledge as well as increase HIV testing and skills for HIV prevention. Peer education is documented as a successful strategy in addressing sexual health issues such as HIV and STI prevention [32]–[35].

Several HIV misconceptions were pervasive in this sample, but there were three particular items related to HIV testing and HIV transmission that were answered incorrectly, which is similar to findings in other studies among college students [9],[10],[12],[20]. Nearly seventy percent of the current sample erroneously believed that they could get tested for HIV one week after a sexual encounter and get an accurate HIV test, which is considerably higher than the seven percent and 12.1 percent found in prior studies [9],[36]. Such a gap could be addressed by partnering with campus care center to develop materials that can be distributed as pamphlets or short messages via social media or that can be infused in general education courses and health related courses.

Additionally, nearly 60 percent of the sample was not aware that latex condoms are more effective against HIV transmission than natural skin condoms. This is significantly higher than the 20 percent [37] and four percent [38] found in previous studies of participants who believed the same misconception. A potential intervention can address such a misconception through sexual health sessions that demonstrate different types of condoms to help students distinguish between latex and natural skin condoms. This can be done in partnership with a campus care center, local health department, student organizations and peer educators.

Nearly half of the sample also erroneously believed that all pregnant women infected with HIV would deliver infected newborns. HIV positive pregnant students may be deterred from seeking prenatal care for fear of being stigmatized for passing HIV to their newborns. HIV stigma is a major deterrent to the prevention of mother to child transmission [39],[40]. Potential interventions can address this misperception by providing information on the efficacy of anti-retroviral medication in reducing the likelihood of a child being born HIV positive [41],[42]. While knowledge of HIV infection may not ultimately lead to perception of transmission risk or change in risky behavior and/or uptake of prevention strategies, it is critical to examine HIV knowledge levels, identify gaps and implement contextually appropriate educational and skill enhancing HIV prevention approaches.

### 4.2. HIV stigma

With regard to HIV stigma, the overall stigma levels in this study were generally low. About 30 percent of participants were afraid of PLWHA and blamed them for acquiring HIV. This rate was lower than 49 percent of participants in Badahdah and Sayem [16] study who blamed those who are HIV infected. However, Djibuti, Zurashvili, Kasrashvili and Berg [43] found that almost 92 percent of the participants had stigmatizing attitudes towards those who are HIV positive. Authors [43] recommended disseminating HIV knowledge including life skills education that can lead to a reduction in HIV stigma as well as enhance HIV prevention. Despite the overall stigma levels being low in the current study, there is still a need to address HIV stigma against PLWHA. One way is to increase knowledge of HIV transmission to ensure that those who aren't infected have adequate knowledge that will mitigate fear of interacting with those infected. Documented evidence supports that people with high levels of HIV knowledge tend to have low levels of HIV stigma [10],[15],[19].

Gender and sexual orientation also had a significant association with HIV stigma against PLWHA. More females had low levels of stigma while heterosexual students had higher level of stigma. Badahdah and Sayem [16] also found that female participants had more positive attitudes towards those living with HIV compared to male participants. Some studies supported the notion that gender difference in empathy are higher among women than men [44]–[46], while others found that female participants had high levels of HIV stigma compared to males [47]. To address HIV stigma, novel strategies need to be employed to enhance HIV prevention behaviors. For example, Moore, Onsomu and Abuya [48] utilized entertainment education (films) as a novel tool to address HIV stigma among college students by engaging them in conversations to address stigmatizing situations highlighted in the film. Such “edutainment” interventions may resonate with this study's priority group.

Findings from this study indicate that more HIV prevention education is needed among university students as increased education reduced stigma. However, this has to be addressed with nuance. It is clear from study findings that there is a need to have more HIV education and skill building strategies to enhance HIV risk susceptibility and uptake of preventive behaviors. Such coordinated efforts are critical due to various reasons. College students lack basic health education knowledge, so more needs to be done at the state level to adopt K-12 health education standards [49] that highlight the need for students to understand health promotion, disease prevention and the need to practice health-enhancing behaviors [50]. This would allow for all students to at least receive a minimum set of health education standards before they graduate from high school. Second, it is imperative that students receive HIV prevention education at the university level using strategies that are contextually relevant (e.g. media, text messages, or edutainment). Perhaps universities need to consider requiring a basic health education class for all freshmen or utilize various communication and message delivery strategies such as campus locations (i.e. campus care centers, student union, and library), website, and/or social media. Research indicates that even brief educational interventions can significantly increase HIV knowledge and reduce stigma [19],[51], so interventions do not need to be complicated; they just need to be tailored to meet the needs of the students.

There are various brief education interventions that have proven to be effective in addressing HIV transmission and can be adapted to meet the needs of the priority population. For example, *AMP!* (Arts-based, Multiple component, Peer-education) [52] program, which was initially developed for high school students, utilizes, “interactive theater-based scenarios developed by trained college undergraduates to deliver messages addressing HIV/STI prevention strategies, healthy relationships, and stigma reduction towards people living with HIV/AIDS” [52]. This program was deemed effective in producing changes in attitudes, knowledge and stigma among its participants in comparison to those who participated in a regular health education course [52]. Secondly, a collaborative partnership with campus care providers and neighboring health institutions that interact with students when conducting HIV testing and counseling, could be developed. The primary goal would be to provide students with additional information when they get tested to minimize HIV testing misconceptions as well as empower them to acquire skills that enhance HIV prevention.

### 4.3. Limitations

The primary limitation is that the data were collected via self-report. It is not possible to assess the honesty of the participants, especially regarding sensitive topic areas such as HIV/AIDS; however, that is an innate limitation in self-report research. In addition, convenience sampling was used to recruit students to take the survey during class sessions as well as by intercepting students at high-traffic areas of the university campus such as the entrance of the main student center. It is possible that the individuals who chose to participate in this study were inherently different from those who chose not to participate. Finally, the sample size of the study is small, but that was because this was a convenience sample to help develop a targeted intervention. Therefore, generalizability beyond the study sample should be done with caution. Study findings can be utilized to develop a larger comparative study that will also include other similarly sized institutions.

## 5. Conclusion

HIV-related knowledge and HIV-related stigma among university students is well documented. It is apparent from the results an interactive comprehensive sexual health intervention that has been effective in other settings can be adopted and tailored to enhance HIV prevention among college students and minimize stigma towards HIV positive individuals.

Additionally, there is a need to coordinate with campus care providers and neighboring health institutions, who interact with students when conducting HIV testing and counseling, to provide students with adequate and accurate information to minimize HIV testing misconceptions. Finally, it is critical to engage diverse peer health educators in the development of HIV prevention education modules tailored to meet the needs of the diverse student population who maybe infected or affected by HIV.
